# Integrative Oncology: Best of Both Worlds—Theoretical, Practical, and Research Issues

**DOI:** 10.1155/2013/383142

**Published:** 2013-11-27

**Authors:** Holger Cramer, Lorenzo Cohen, Gustav Dobos, Claudia M. Witt

**Affiliations:** ^1^Department of Internal and Integrative Medicine, Kliniken Essen-Mitte, Faculty of Medicine, University of Duisburg-Essen, 45276 Essen, Germany; ^2^Department of General Oncology and the Integrative Medicine Program, The University of Texas MD Anderson Cancer Center, Houston, TX 77030, USA; ^3^Charité Universitätsmedizin Berlin, Institute for Social Medicine, Epidemiology and Health Economics, 10M7 Berlin, Germany; ^4^University of Maryland School of Medicine, Center for Integrative Medicine, Baltimore, MD 21201, USA

## Abstract

More and more cancer patients use complementary therapies. As the majority of patients do not disclose their use of complementary therapies to their oncologists, they expose themselves to possible detrimental effects from the therapies due to drug interactions. To meet the needs of patients and health care professionals on valid information on complementary therapies, the collaborative research project “Competence Network Complementary Medicine in Oncology—KOKON”, an interdisciplinary network for complementary medicine research in oncology, was established. Moreover, Integrative Oncology, a combination of conventional and evidenced-based complementary therapies delivered using a comprehensive approach, is now increasingly used in the United States and Europe. A variety of different Integrative Oncology models have been established worldwide including an expert-based model at the Kliniken Essen-Mitte, Essen, Germany and a patient-centered, evidenced-based approach at The University of Texas MD Anderson Cancer Center. Both models are briefly reviewed. More research is needed and Comparative Effectiveness Research that places strong emphasis on the comparison of different treatment options in usual care settings by including more heterogeneous patients, using less standardized treatment protocols, and measuring patient-centered outcomes would provide useful information for decision-making. To improve the quality of care and research in Integrative Oncology, sustainable financial models for Integrative Oncology and more funding for research are needed.

## 1. Integrative Oncology

With about 12.7 million new cases and 7.6 million deaths in 2008, cancer is the leading cause of death worldwide [1, 2]. While survival rates are increasing [3, 4], cancer diagnosis and treatment are still often associated with physical and psychosocial impairments. While more than half of all cancer patients report fatigue as a problem [5, 6], one out of three cancer patients suffers from a mood disorder at some point in their cancer care trajectory [7]. The most common complaints in cancer patients concern pain, fatigue, depression, and anxiety [5, 8].

Complementary and alternative medicine (CAM) is used by about 40% of all cancer patients to manage symptoms related to their disease, with varying estimates usually based on the definition of CAM [9–12]. Patients normally do not expect these approaches to cure their disease but mainly use them to strengthen their immune system, relieve pain, or manage treatment-related side effects [13]. As patient survival rates are increasing, those needs of cancer survivors that go beyond the mere alleviation of symptoms are becoming more important for oncologists, psycho-oncologists, and other oncology professionals. Many complementary therapies aim to treat patients in a more comprehensive manner and thus are also concerned with the patient's psychological and spiritual needs. Complementary oncological therapies are classified by the Concerted Action for Complementary and Alternative Medicine Assessment in the Cancer Field (CAM-Cancer) as

1. alternative medical systems (e.g., homeopathy, traditional Chinese medicine)
2. biologically based practices (e.g., herbs, vitamins, and food)
3. energy medicine (e.g., reiki)
4. manipulative and body-based practices (e.g., massage)
5. mind-body medicine (e.g., meditation, yoga, and progressive muscle relaxation) [14].

Complementary therapies are now even included worldwide in the curricula of many medical schools [15, 16], including such prestigious schools as Stanford University Medical School, the Harvard Medical School, and the Charité University Medical Center in Berlin, Germany, to name a few. In fact, the US-based Consortium of Academic Health Centers for Integrative Medicine has over 50 academic centers as part of their membership. In addition, integrative oncology is now being implemented in various institutions such as the Memorial Sloan-Kettering Cancer Center, The University of Texas MD Anderson Cancer Center and other institutions in the USA, Canada, and Europe [17, 18].

While alternative therapies are used instead of conventional medicine, complementary therapies are used in addition to conventional methods. Conventional clinicians are more supportive of the use of complementary therapies than alternative therapies as in the former the patients are not foregoing treatments with known clinical benefit. However, simultaneous administration of complementary and conventional therapies always bears the risk that the complementary treatment will interfere with the standard treatment, for example, in the form of drug interactions, partially with incalculable outcomes. Additionally, the majority of patients do not disclose their use of complementary therapies to their health care team mainly due to lack of inquiry; patient's anticipation of the doctor's disapproval, disinterest, or inability to help; and patient's perception that disclosure of CAM use is irrelevant to their conventional care [19]. Thereby, they expose themselves to possible adverse effects. These are especially due to the possible interactions of complementary treatments with chemotherapy, radiotherapy, endocrine treatment, or other targeted therapies. Integrative oncology is a combination of conventional with complementary therapies that have been shown to be safe and effective [20]. In contrast to alternative or complementary approaches, Integrative oncology aims to combine the best practices of conventional and complementary oncological therapy (the “best of both worlds”). Thereby, conventional and complementary therapies are united into one comprehensive, patient-centered approach in order to maximize safety and efficacy. As both treatment approaches are administered from members of the same team, occasional interactions between treatments can be recognized and the best possible outcomes to meet patients' needs can be ensured [21, 22].

## 2. The Relevance of Valid Patient Information

The majority of cancer patients today want detailed information about their cancer diagnosis, prognosis, and treatment options [23], and information seeking has been demonstrated to play a critical role in individuals' efforts to cope with the disease [23]. The benefits of information for cancer patients include increased involvement in decision making and greater satisfaction with treatment choices [24], reductions in psychological distress, and improved communication [25]. Patients most frequently seek treatment-related information, such as treatment options and side effects [26]. Cancer patients often expect important benefits from complementary medicine. Irrespective of how well informed patients are, a substantial percentage of patients still need additional information, often about topics such as food, diet, and complementary medicine treatment options; relatively often, even patients already using complementary medicine felt a need for additional information [27]. Likewise, they perceived important unmet needs for readily accessible, credible, relevant sources of complementary medicine information. There is a strong need for more efficient, systematic, and trustworthy information. Typically, patients have located information under conditions of great stress and uncertainty and would welcome improvements in that process [28].

To meet the needs of patients and health care professionals, the German Cancer Aid (Deutsche Krebshilfe) is currently funding the collaborative research project “Competence Network Complementary Medicine in Oncology-KOKON”. KOKON is organized as a closely linked cluster of seven work packages with the overall aim to develop, implement, and evaluate an interdisciplinary network for complementary medicine research in oncology. The work packages and analyzes the needs for and offers information for cancer patients and health care professionals around complementary medicine and is working to develop a web-based information platform. Furthermore, KOKON is engaged in the development, implementation, and evaluation of a medical expert consulting service for complementary medicine as well as training programs for health care professionals and cancer survivors. All methodological approaches are supported and harmonized by a methodology center, in order to guarantee high scientific quality. A coordination center ensures broad cooperation within KOKON and a multidisciplinary board of external experts assures supervision of the research project.

## 3. Clinical Models for Integrative Oncology

A substantial number of cancer patients use complementary therapies to manage symptoms related to their disease or conventional treatment [12]; the prevalence of complementary therapies use has almost doubled from the 1970s until the 2000s [12]. The majority of patients use CAM methods indiscriminately and without informing their oncologists [11], exposing themselves to possible detrimental effects, especially due to interactions with their conventional treatments. Therefore, integrative oncology programs have been established that can advise patients about both the benefits and the possible negative effects of complementary therapy treatments. A variety of different models are used worldwide [18]; two such models are briefly introduced below. Similarities and differences between the 2 models are shown in Table 1. A more detailed comparison of the 2 models can be found in [29].

### 3.1. Integrative Oncology for Breast Cancer Patients in Germany: An Expert-Based Model

Since the beginning of 2010, two departments of the Kliniken Essen-Mitte, academic teaching hospital of the University of Duisburg-Essen, the Department of Senology/Breast Center (medical director: PD Dr. S. Kümmel), and the Department of Internal and Integrative Medicine (Professor Dr. G. Dobos) have been cooperating in providing integrative oncology for breast cancer patients. The treatment of each patient is organized according to an individualized treatment plan based on the current literature and treatment guidelines [30]. These treatment plans are formulated based on detailed analyses of a patient's case and the results of their individual tumor conferences. During this, the integrative oncology team, consisting of physicians and complementary medicine therapists, reviews current research literature and guidelines relevant to the specific breast cancer patient. For this purpose, SenoExpert, a special database for breast cancer patients, was developed that is continually updated by scientists, physicians, and therapists who regularly review the medical literature and screen it for new guidelines. SenoExpert aims to make the current guidelines and scientific evidence readily available to physicians responsible for routine care. In addition, experts discuss anonymized breast cancer cases at regular intervals during online conferences. Besides conventional oncological treatment every patient has the opportunity to attend consultations with complementary therapies physician and mind-body medicine therapists, specially trained health professionals with expertise in clinical psychology, nutrition, exercise, and psycho-oncology. Treatment options include acupuncture, phytotherapy, gua sha therapy, and cupping massage while mind-body medical therapies include physical exercise, yoga, mindfulness, training on coping strategies, and nutrition in order to support patients in coping with their diseases and treatments [30]. Since October 2010, the number of CAM treatments provided is increasing each year (Table 2) [30].

As the evidence base supports that mind-body medicine is effective for improving health-related quality of life, psychological, and physical health in cancer patients [31–33], mind-body medicine is a crucial part of integrative oncology at the Department of Complementary and Integrative Medicine, Kliniken Essen-Mitte [30]. To deepen and consolidate the knowledge and skills acquired during their inpatient stay, patients are offered participation in an 11-week mind-body medicine day care clinic subsequent to their inpatient stay [34]. This program is open for but not limited to patients who underwent inpatient treatment at the Kliniken Essen-Mitte. So far, about 1500 cancer patients have participated in this program [34]. Based on the Mindfulness-Based Stress Reduction (MBSR) Program developed by Kabat-Zinn at the University of Massachusetts [35, 36] and the mind-body medicine cancer program of the Benson-Henry Mind/Body Medical Institute at Harvard Medical School [37], the day care program integrates cognitive therapy, meditation, yoga, exercise training, nutritional lectures in a teaching kitchen, and self-care strategies (e.g., cupping massage [38], hydrotherapy). In addition, during weekly group medical rounds, patients can discuss their current medical status and their progress over the course of the program with an integrative oncologist. The effectiveness of the day care program has already been investigated in a number of clinical trials [39–41].

Beyond inpatient and outpatient care, the Breast Center and the Department of Internal and Integrative Medicine, Kliniken Essen-Mitte, work closely together to improve the quality of integrative oncology training: a curriculum for advanced training of physicians has been developed and an official qualification in integrative oncology is currently being accredited. Moreover, in order to improve the evidence base for including complementary therapies in treatment plans for cancer patients, both departments are involved in the development of medical guidelines in 2011 and 2013, in cooperation with the study group for gynecologic oncology (AGO).

### 3.2. The Integrative Medicine Program at MD Anderson Cancer Center

The Integrative Medicine Program at MD Anderson focuses on creating a comprehensive and integrative care plan that addresses the whole person with a cancer diagnosis. The Program's mission focuses on three areas: clinical delivery, education, and research. The main objectives of these areas are as follows: (1)  *Clinical*: to provide the highest quality integrative medicine therapies to patients and their families using a patient-centered approach. The therapies are provided in concert with mainstream care to manage symptoms, relieve stress, and enhance quality of life; (2)  *Education*: offer reliable information on integrative medicine interventions to patients, families, and medical staff of MD Anderson; and (3)  *Research*: advance knowledge on the outcome and effectiveness of integrative therapies through peer-reviewed, mixed-methodology research.

The Integrative Medicine Program at MD Anderson is an important component of the mission to treat the whole person—from prevention and treatment through survivorship. Ongoing research is examining intervention programs and treatments that can improve quality of life and clinical outcomes. Educational programs provide information to our faculty, staff, students, trainees, and the public about complementary and integrative medicine (CIM) approaches.

The Integrative Medicine Center, the clinical delivery component of the program, offers integrative oncology consultation with an oncologist with CIM training and focuses on the use of CIM throughout the cancer care continuum. These consultations are initiated by the primary oncologist similar to a referral to another specialty. During this consultation, the integrative oncologist reviews details regarding the cancer history and treatment plan, gaining an understanding of each patient's needs and reasons for CIM interest. This discussion ranges from providing expertise in natural products, including nutritional supplements, vitamins and herbs, to incorporating mind-body techniques such as music therapy and meditation. Other topics may focus on managing pain, stress and anxiety, and other symptoms resulting from illness and/or treatment side effects. The goals are to help the patient obtain optimal health and healing through a treatment plan that is comprehensive, integrative, personalized, evidence-based, and safe. Close collaboration with the primary oncology team is essential and treatment plans are placed within the context of the patient's goals of care. Challenging cases are discussed at a weekly multidisciplinary team meeting to develop individualized treatment plans. The treatment plans commonly involve multiple health practitioners including dieticians, meditation instructors, chaplains, physical/occupational therapists, acupuncturists, massage therapists, yoga instructors, music therapists, and others. Both individual and group programs are provided to patients and caregivers. Individual services include consultation with an integrative oncologist, dietary and exercise consultations, meditation/psychological consult, acupuncture, massage, and music therapy. Group programs include a number of different mind-body and movement-based practices (e.g., meditation, yoga, tai chi, qigong, and Pilates), nutrition classes, music therapy, expressive arts, support groups, and more. An inpatient consult services is also available.

## 4. The Challenges and the Future of Clinical Research

Integration should be based on evidence and the gap between the usage in cancer patients and the available clinical evidence is still large. Furthermore, there is an ongoing discussion in the field of medicine about which type of evidence might be the most suitable. Despite the decades of effort to provide solid evidence of the efficacy and effectiveness of interventions, results have yielded relatively little evidence to support health care decisions. For stakeholders (e.g., patients, payers, clinicians, and policy makers), the available evidence on specific treatment effects often fails to provide clarity for decision makers when they are confronted by choices between and among a variety of options for a heterogeneous patient population. The rigor in randomized studies, wherein patients with comedication and comorbidities are often excluded and treatments are highly standardized, has largely provided results that are poorly generalizable.

Comparative effectiveness research (CER) is a more recent development in clinical research that places strong emphasis on the comparison of different treatment options in usual care settings by including more heterogeneous patients, using less standardized treatment protocols, and measuring patient-centered outcomes [42]. The Institute of Medicine in the US identified cancer as one priority for CER [43] and a strategic framework for how to perform CER in the field of oncology was developed by the Center for Medical Technology Policy [44]. In general, a broad spectrum of methods—from registries to randomized trials—can be applied to CER. The strategic framework for CER in the field of complementary and integrative medicine concluded that CER should be prioritized and CER study designs should focus on effectiveness rather than on efficacy [45]. “Efficacy” refers to “the extent to which a specific intervention is beneficial under ideal conditions,” whereas “effectiveness” is a measure of the extent to which an intervention, when deployed in the field in routine circumstances, does what it is intended to do for a specific population [46].

Integrative oncology is a field particularly well suited for CER, because it often deals with the use of both conventional and complementary treatments and points to patient populations that usually have comorbidities and use a broader range of medications. By fostering study designs that emphasize broader inclusion criteria, more wide-ranging outcomes (including quantitative and qualitative outcomes), subgroup analyses, stakeholder input, usual care settings, and flexible treatment protocols, CER has the potential to yield important evidence for decision makers in the field of integrative oncology.

## 5. Conclusions

Complementary therapies are increasingly requested by cancer patients and integrative oncology programs are established worldwide to serve their needs. In order to improve the quality of care for cancer patients, more clinical research is needed to investigate effective and safe combinations of conventional and complementary treatments. In particular, more funding for research that is relevant for clinical decision making is urgently needed. Also, in order to establish clinical guidelines, a systematic review of the current literature is necessary. To improve the knowledge of clinicians working in integrative oncology, the development of curricula for integrative oncology summarizing the evidence-based procedures that are safe and effective is urgently needed. To facilitate the extensive worldwide implementation of integrative oncology, sustainable financial models for clinical care are needed.

## Figures and Tables

**Table 1:** Similarities and differences between the Integrative Oncology for Breast Cancer Program, Essen, Germany and the Integrative Medicine Program, Houston, USA [29].

	Integrative Oncology for Breast Cancer Program, Essen	Integrative Medicine Program at MD Anderson Cancer Center, Houston
Center	Breast cancer center offering conventional and integrative medicine	Integrative medicine referral center open to all conventional departments
Setting	Inpatient and outpatient	Inpatient and outpatient
Philosophy	Holistic and patient-centered approach	Holistic and patient-centered approach
Treatments	Mind-body medicine, acupuncture, massage therapy, naturopathic treatments, nutrition, exercise, phytotherapy	Mind-body medicine, acupuncture, massage therapy, nutrition, exercise, music therapy, expressive arts, support groups
Professional team	Oncologists, physicians specialized in complementary medicine, mind/body medicine instructors	Physicians specialized in both oncology and complementary medicine, dieticians, meditation instructors, chaplains, physical therapists, acupuncturists, massage therapists, yoga instructors, music therapists
Research	Implementation of an evidence-based information database (“SenoExpert”)	Well-funded research program and cooperative projects with other departments

**Table 2:** Number of selected complementary and alternative medicine (CAM) treatments provided in the Integrative Oncology for Breast Cancer Program, Essen, Germany in 2011 and 2012.

	2011	2012
Body acupuncture	560	1043
Ear acupuncture	1587	1509
Mind-body medicine (counseling, exercise, diet, relaxation, yoga, qigong)	548	1273
CAM treatments overall	4398	5838
